# Impact of Truncated *O*-glycans in Gastric-Cancer-Associated CD44v9 Detection

**DOI:** 10.3390/cells9020264

**Published:** 2020-01-21

**Authors:** Inês B. Moreira, Filipe Pinto, Catarina Gomes, Diana Campos, Celso A. Reis

**Affiliations:** 1 I3S–Instituto de Investigação e Inovação em Saúde, Universidade do Porto, 4200-135 Porto, Portugal; imoreira@ipatimup.pt (I.B.M.); filipep@ipatimup.pt (F.P.); cgomes@ipatimup.pt (C.G.); 2 IPATIMUP–Institute of Molecular Pathology and Immunology, University of Porto, 4200-135 Porto, Portugal; 3 Faculty of Medicine, University of Porto, 4200-319 Porto, Portugal; 4 Instituto de Ciências Biomédicas Abel Salazar, University of Porto, 4050-313 Porto, Portugal

**Keywords:** CD44, glycosylation, gastric cancer, truncated *O*-glycans

## Abstract

CD44 variant isoforms are often upregulated in cancer and associated with increased aggressive tumor phenotypes. The CD44v9 is one of the major protein splice variant isoforms expressed in human gastrointestinal cancer cells. Immunodetection of CD44 isoforms like CD44v9 in tumor tissue is almost exclusively performed by using specific monoclonal antibodies. However, the structural variability conferred by both the alternative splicing and CD44 protein glycosylation is disregarded. In the present work, we have evaluated the role of *O*-glycosylation using glycoengineered gastric cancer models in the detection of CD44v9 by monoclonal antibodies. We demonstrated, using different technical approaches, that the presence of immature *O*-glycan structures, such as Tn and STn, enhance CD44v9 protein detection. These findings can have significant implications in clinical applications mainly at the detection and targeting of this cancer-related CD44v9 isoform and highlight the utmost importance of considering glycan structures in cancer biomarker detection and in therapy targeting.

## 1. Introduction

Gastric cancer (GC) is one of the major problems in public health worldwide, killing up to nearly one million people worldwide each year [1]. The absence of specific symptoms, together with the use of invasive diagnostic tools, often results in late diagnosis and consequently a lack of an effective treatment [2]. In fact, therapeutic options are limited in GC. Currently, major efforts are focusing on targeting the cells with tumor-initiating capacities, cancer stem cells (CSCs), whose unlimited proliferation potentials are known to drive tumor growth, invasion, metastasis, and resistance to various forms of therapy [3,4].

Among the known CSCs biomarkers, CD44 has unique features that make it one of the most promising tumor markers. CD44 is a transmembrane glycoprotein broadly expressed among different cells and is known as the major receptor for hyaluronic acid (HA) [5]. Previous studies demonstrated that CD44-positive GC cells displayed all basic CSC features and also chemo- and radio-resistance properties, which are likely to account for the resistance of this tumor type to most standard treatment protocols [6].

CD44 is structurally and functionally heterogeneous. Protein heterogeneity results, in part, from alternative splicing of 9 of the total 19 known exons codified in the genome. Exons 6 to 14 can be alternatively spliced and placed between the two constant regions to generate different CD44 variants (CD44v), CD44v2 to v10 [5]. Additional protein heterogeneity is generated by cell-specific glycosylation [7]. CD44 variable isoforms are heavily glycosylated, with a total of nine predicted *N*-glycan sites, two sites of attachment of glycosaminoglycans [5], and 146 predicted *O*-glycan sites [5,8].

Together, alternative splicing and glycosylation make CD44 one of the most variable surface molecules and explain its various cellular functions. Although the roles of CD44v in CSCs remain elusive, there is increasing evidence that they are key molecules in tumor growth and progression. The CD44v8-10 has been claimed to be the predominant CD44 variant found in gastric CSCs from patient tumor samples [9], and its expression was shown to promote resistance against reactive oxygen species (ROS) and contributed to cell proliferation [10]. Particularly, CD44v9, contained in CD44v8-10, is one of the major isoforms in human gastrointestinal cancer [11], and its presence is related to elevated tumor sizes and poor prognosis and survival [12,13]. Moreover, the mechanistic importance of CD44v9 highlights its potential as a promising biomarker and potential target in therapeutic strategies in GC.

Malignant transformation is also accompanied by aberrant glycosylation of proteins. One of the major types of cancer-specific glycosylation is the expression of truncated *O*-glycans, which is associated with poor prognosis and low survival of the patients [14,15] and is highly expressed in most gastric tumors [16,17,18]. In a previous work, CD44 proteins containing the STn structure were detected in both GC tissue and in circulation from matching patient sera [19], as CD44 can be extracellularly cleaved and detected in circulation [20]. The higher levels of serum CD44-STn have been shown to be able to differentiate between patients and healthy subjects [19]. Additionally, in previous work from our group, CD44 was identified in GC cell lines as a potential secreted biomarker with immature *O*-glycans, which was validated in cancer patients′ sera [21]. In the same work, a single *O*-glycosite identified by mass spectrometry in GC patient′s serum was in the v9 exon. Therefore, the focus on this specific CD44 isoform variant combined with cancer-related glycan structures could pave the way for new biomarkers in GC.

Herein, we aim to define the CD44v9 detection in gastric cancer cell line models displaying the *O*-glycosylation machinery resembling the malignant phenotype. The influence of *O*-glycosylation elongation disruption in molecular features of CD44 and CD44v9 is assessed, to determine whether CD44v9 detection in GC cells depends on its glycoprofile. Our work highlights the importance of glycosylation in antigen recognition and further addresses how glycosylation can affect reliability of CD44v expression detection *in vivo* as well as its prognostic value.

## 2. Materials and Methods

### 2.1. Antibodies and Lectins

Monoclonal antibodies (mAbs) 1E3 (anti-Tn; Clausen and Hakomori, Unpublished Data), B72.3 (anti-STn) [22] and 3C9 (anti-T) [23] were purified from hybridoma culture media. CD44 (156-3C11) mAb was obtained from Cell Signalling Technology (Leiden, The Netherlands), CD44v9 (RV3) mAb from Abnova (Taiwan, China), CD44v (CD44v6 – MA54) from Invitrogen (Waltham, MA, USA) and tubulin (DMA1A-9) mAb from Merck (Darmstadt, Germany). 

Secondary antibodies goat anti-mouse and donkey anti-rat Alexa Fluor^®^ 488 and goat anti-mouse Alexa Fluor^®^ 594 were purchased from Invitrogen; horseradish peroxidase-conjugated goat anti-mouse IgG from Jackson ImmunoResearch (Cambridge, UK) and goat anti-rat IgG from Santa Cruz Biotechnology (Santa Cruz, CA, USA).

### 2.2. Cell Culture

The gastric carcinoma cell lines MKN45 (Lauren diffuse-type) and AGS (Lauren intestinal-type) [24] were obtained from the Japanese Collection of Research Bioresources and ATCC, respectively. The MKN45 and AGS SimpleCell models (MKN45 SC and AGS SC) were obtained by targeting the *C1GALT1* (COSMC) gene using zinc-finger nuclease precise gene editing as previously described [21,25]. Furthermore, MKN45 cell line was stably transfected with the full-length human *ST6GALNAC1* gene (MKN45 ST6) or the corresponding empty vector pcDNA3.1 (MKN45 MOCK) as previously described [26,27]. The MKN45 WT/SC and AGS WT/SC cell lines were cultured in RPMI 1640 GlutaMAX™, HEPES medium (Gibco, Waltham, MA, USA). The MKN45 MOCK/ST6 cell lines were cultured in RPMI supplemented with 0.5 mg/mL of G418 (Invitrogen). All media were supplemented with 10% heat-inactivated FBS (Biowest, Riverside, MO, USA). All cells were grown at 37 °C in an atmosphere of 5% CO_2_.

### 2.3. Immunofluorescence

Cells were seeded in 96-well plates or in 12-well plates coverslips and were left in the incubator untreated or exposed to DMSO or drug treatment: *O*-glycosylation inhibitor Benzyl 2-acetamido-2-deoxy-α-D-galactopyranoside (B4894) for 48 h or proteasome inhibitor MG132 (M8699) for 1 h, both purchased from Sigma-Aldrich (St. Louis, MO, USA). After fixation with paraformaldehyde, coverslips were permeabilized with PBS 0.2% Triton X-100 (Merck Millipore, Burlington, MA, USA). Nonspecific binding was blocked with 10% BSA in PBS for 30 min at room temperature. Cells were stained with primary antibodies overnight at 4 °C. Antibody concentrations: CD44 at 1:400; CD44v9 at 1:100; CD44v at 1:200; Tn, STn, and T undiluted. Secondary antibodies anti-mouse and anti-rat Alexa Fluor^®^ 488 and anti-mouse Alexa Fluor^®^ 594 were used at 1:500. Nuclear stain was performed with DAPI (Sigma-Aldrich). Cells were washed with PBS, and images were acquired using Zeiss AxioImager Z1 (Carl Zeiss, Oberkochen, Germany) or IN Cell Analyzer 200 (GE Healthcare, Little Chalfont, UK).

### 2.4. Flow Cytometry

MKN45 cells were seeded in 6-well plates 48 h before analysis. For cell cycle synchronization, cells were cultured in serum starvation for 24 h before replacing the serum in the media. On the day of analysis, cells were detached using Gibco^®^ Versene solution (ThermoFisher, MA, USA) on indicated timepoints, spun down at 300 g for 5 min at 4 °C, and resuspended in Fixable Viability Dye APC-Cy7 (FVD 1:2000, ThermoFisher). For permeabilization, cells were fixed with 2% PFA and then treated with 0.5% Saponine (Sigma-Aldrich). For cell cycle analysis, cells were fixed in 70% ethanol. Afterwards, they were stained with primary antibodies CD44 (1:100), CD44v9 (1:100), or STn (undiluted) for 30 min on ice. Cells were washed and incubated with secondary antibodies anti-mouse or anti-rat (1:200) for 30 min on ice in the dark. For the cell cycle analysis, cells were stained with DAPI (10 μg/mL) for 20 min at room temperature in the dark. Samples were analyzed on a FACS Canto II Flow cytometer (BD, San Diego, CA, USA).

### 2.5. RNA Isolations and Real-time (RT)-qPCR

Cells were grown in 6-well plates and lysed with TRI Reagent (Sigma-Aldrich), and total RNA was extracted according to the manufacturer. cDNA was synthesized using 2000 ng of RNA diluted in nuclease-free H_2_O. Random primers (Thermo Scientific) and deoxynucleotides (NZY Tech) were added, and the samples were heated at 65 °C for 5 min, followed by 1 min on ice. The reaction mix was added, containing 5X SSIV buffer, DTT reagent, RNase OUT recombinant RNase Inhibitor and SuperScript ^®^ IV Reverse Transcriptase (Invitrogen). Primer sequences were as follows (5′ to 3′): total CD44 isoforms (CD44total PCR) forward (TGGGTTCATAGAAGGGCATG) and reverse (ATTTGGGGTGTCCTTATAGG), CD44 constant region (CD44total RT-qPCR) forward (TGGGTTCATAGAAGGGCACG) and reverse (TGCTGGGGTAGATGTCTTCAGG), CD44v9 forward (5′ AGCAGAGTAATTCTCAGAGC 3′) and reverse (TGCTTGATGTCAGAGTAGAA), CD44v8-10 forward (CGCTTCAGCCTACTGCAAATCC) and reverse (GTCTTAGCTGAGGTCACTGGG). Relative gene expression was normalized to β-actin (forward: AGAAAATCTGGCACCACACC; reverse: TAGCACAGCCTGGATAGCAA), and ∆∆ CT was performed. Two independent experiments with two technical replicates were performed.

### 2.6. Western Blotting

Cells were seeded in 75 cm^2^ flasks or 6-well plates and were left in the incubator untreated or exposed to DMSO or drug treatment. Following drug treatment, cells were washed twice with ice-cold PBS, scraped into lysis buffer 17 (R&D Systems, McKinley Place, Shorewood, MN, USA) supplemented with 1 mM sodium orthovanadate, 1 mM phenylmethanesulfonylfluoride, and protease inhibitor cocktail (Roche, Basel, Switzerland). Tubes containing cell lysates were held on ice for 30 min, vortexing every 5 min, and then spun at 17,000x g for 15 min at 4 °C. The supernatant was moved to a clean tube, the protein concentration was measured by BCA Assay (Bio-Rad, Berkeley, CA, USA), and a total amount of 25 μg of protein extract was used in each experiment. Samples were mixed with 4X Laemmli buffer, 10X DTT and run on 4–15% SDS-Page gels. After transferring to nitrocellulose membranes, they were blocked with 5% milk in TBS-tween for 1 h and incubated overnight with primary antibodies at 4 °C. Antibody concentrations: CD44 at 1:1000, CD44v9 at 1:1000, tubulin at 1:10000 and T undiluted. Secondary antibodies anti-mouse and anti-rat were used at 1:5000 or 1:2000, respectively. The signal was detected with ECL™ Western Blotting detection reagents (Amersham™, GE Healthcare) and high-performance chemiluminescence films, exposed in a Hypercassette™ and revealed and fixed with developer and fixer solutions, respectively. Densitometry was performed with GS-800 Calibrated Densitometer (Bio-Rad).

### 2.7. Statistical Analysis

Data were analyzed with GraphPad Prism 6.0 software (GraphPad, San Diego, CA, USA). Quantitative data were expressed as the mean ± SD of at least three replicates. The significance of differences between the values was assessed using Student′s *t*-test. *p*-values of <0.05 were considered significant.

## 3. Results

### 3.1. Glycoengineered Gastric Cancer Cell Lines Enable In Vitro Study of O-glycosylation Truncation

**Figure 1:**
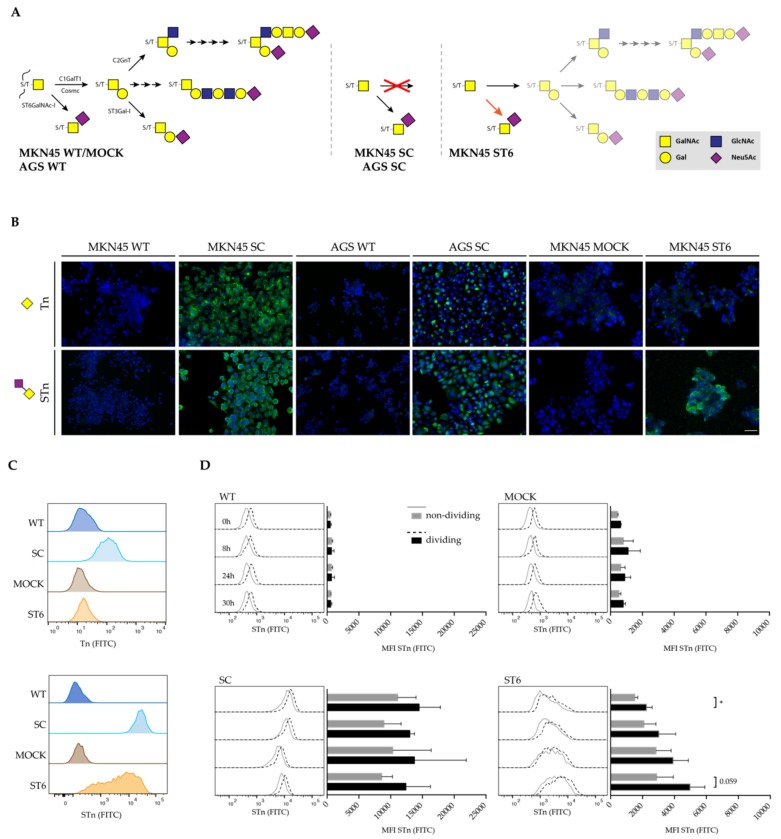
Truncated *O*-glycans in gastric cancer cell line models. (**A**) Schematic representation of the *O*-glycosylation pathway of glycoengineered gastric cancer cell line models. (**B**) Immunofluorescence analysis of truncated *O*-glycans (Tn and STn) in gastric cancer cell line models of *O*-glycan truncation (MKN45 SC, AGS SC, and MKN45 ST6) and their respective control counterparts (MKN45 WT, AGS WT, and MKN45 MOCK). Nuclei were stained with DAPI and are shown in blue and *O*-glycan structures in green. The scale bar corresponds to 50 μm. (**C**) Histograms of Tn and STn fluorescence signal in flow cytometry in MKN45 glycoengineered cell line models, SC and ST6, as compared to their control cell lines, WT and MOCK. Data are representative of three independent experiments. (**D**) Median fluorescence intensity (MFI) quantification of fluorescence signal related to STn expression in MKN45 glycoengineered cell models, SC and ST6, as compared to their control counterparts, WT and MOCK, according to cell cycle progression. The analysis resulted from different incubation times (0 h, 8 h, 24 h, and 30 h), after cell cycle synchronization through 24 h of serum starvation. All MFI levels were subtracted from the negative control intensity. Data are representative of at least three independent experiments. * *p* < 0.05.

In order to evaluate *in vitro* gastric cancer cell models displaying *O*-glycosylation features commonly observed in gastric tumors, previously generated genetic engineered models of *O*-glycosylation truncation were used: (i) SimpleCell models (MKN45 SC and AGS SC) were developed using zinc finger nucleases (ZFNs) to disrupt *COSMC* [21,25]; (ii) stably transfected *ST6GalNAc-I* overexpressing models (MKN45 ST6 cells) and a mock control (MKN45 MOCK cells) [26] (Figure 1A). Both models overexpress truncated *O*-glycans, as can be accessed using monoclonal antibodies detecting Tn and STn structures (Figure 1B,C). Immunofluorescence (IF) analysis of the SC models showed similar detection of Tn and STn, whereas the ST6 displayed enhanced expression of STn with virtually no expression of Tn (Figure 1B). Cell surface expression evaluation by FACS analysis revealed that the SC model displays an homogenous profile of Tn-/STn-expressing cells, whereas the ST6 model showed an heterogenous profile (Figure 1C). This finding is in agreement with the fact that the ST6 model allows for partial *O*-glycan extension (Figure 1A) [26,28,29]. In addition, further characterization of STn-expression dependency on cell cycle progression was analyzed. Evaluation of synchronized cells in G1 by serum starvation showed that STn surface expression is increased in dividing cells in the ST6 model when compared to nondividing cells (Figure 1D). This result agrees with Golgi apparatus growth and increased transport in premitotic stages of the cell cycle [30]. In the SC model, and since the STn expression is homogenous, we were not able to find differences between nondividing and dividing cells. These results demonstrate that the selected glycoengineered cell lines are good models to study *O*-glycan truncation in gastric cancer.

### 3.2. Total CD44 and CD44v9 Expression in Gastric Cancer Cell Line Models of O-glycosylation Truncation

**Figure 2:**
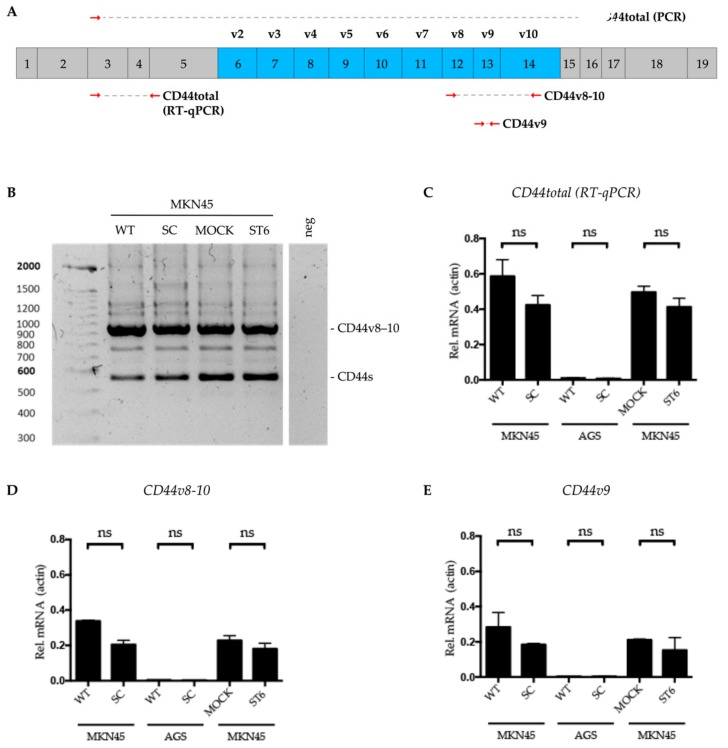
*CD44* gene expression analysis in gastric cancer cell line models. (**A**) Primer scheme for *CD44* isoform analysis through PCR and RT-qPCR. → forward primer; ← reverse primer. (**B**) Analysis of the total set of *CD44* isoforms expressed in gastric cancer cell line models of *O*-glycan truncation (MKN45 SC and ST6) and their control counterparts (MKN45 WT and MOCK). Expected molecular weights are matched with *CD44* transcript isoforms. (**C**–**E**) Analysis of the mRNA expression of *CD44* isoforms by RT-qPCR: total *CD44* (**C**) *CD44v8-10* (**D**) and *CD44v9* (**E**). Results were normalized to the actin transcript expression. Analysis were performed in two biological replicates with two technical replicates each and are shown as average ± SD. ns = non significant.

CD44 expression has been associated with gastric cancer disease progression and aggressiveness [12,31,32], revealing its importance in these types of malignancies. In order to evaluate the impact of truncated *O*-glycans cellular profile, we first determined the product of alternative splicing of the *CD44* gene in the presented models. Primers were designed so all variants would be amplified on the cDNA from total RNA extracts (Figure 2A red arrows). The PCR products for the several isoforms were separated according to the molecular weight in an agarose gel electrophoresis, and the band sizes were matched with *in silico* analysis of the mRNA after alternative splicing. The *CD44v8-10* transcript, a *CD44v9*-containing isoform, is the most represented in the MKN45 cell lines (Figure 2B). Also, the global *CD44* isoforms profile was not altered in the truncated *O*-glycosylation models, when compared to the parental cell line, MKN45 WT (seen through band intensity measurements, data not shown). Next, we evaluated the expression of the total *CD44-* and *CD44v9*-containing isoforms by quantitative RT-qPCR, namely *CD44v8-10* and *CD44v9*. There are no significant differences regarding the expression of the transcripts analyzed, between the *O*-glycosylation truncation models and their control counterparts (Figure 2C–E). The relative presence of *CD44* isoforms in the total pull of *CD44* transcripts is also not altered between the models. 

**Figure 3:**
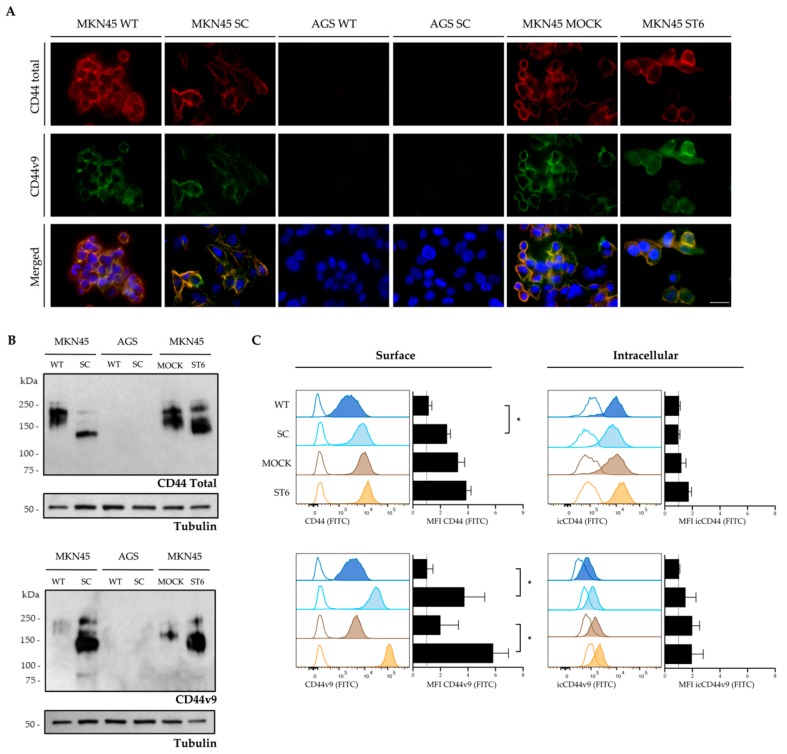
CD44v9 in a truncated *O*-glycan context. (**A**) Immunofluorescence analysis of CD44 isoforms (total CD44 and variant 9) in gastric cancer cell line models of *O*-glycan truncation (MKN45 SC, AGS SC, and MKN45 ST6) and their respective control counterparts (MKN45 WT, AGS WT, and MKN45 MOCK). Nuclei were stained with DAPI and are shown in blue, CD44 total in red and CD44v9 in green. The scale bar corresponds to 25 μm. (**B**) Western blot analysis of CD44 total and CD44v9 in glycoengineered gastric cancer cell lines and their respective control counterparts. (**C**) MFI quantification of the flow cytometry analysis of surface or intracellular CD44 isoforms (total CD44 and CD44v9) of MKN45 glycoengineered cell models as compared with their control counterparts. Negative controls are shown in dotted lines and indicate the samples incubated with the secondary antibody only. All MFI levels were subtracted from the negative control intensity and normalized to the MKN45 WT cell line. Data is representative of at least three independent experiments. * *p* < 0.05.

We further evaluated the receptor expression by immunofluorescence, western blot, and flow cytometry using specific mAbs directed to either total CD44 protein or CD44v9 (Figure 3). Double immunofluorescence analysis revealed that MKN45 models express both total CD44 and CD44v9, whereas they were not detected in the AGS models (Figure 3A). Protein extracts were used to perform a western blot analysis of the same cell line models (Figure 3B). All the MKN45 models showed CD44 and CD44v9 presence, in agreement with previous data, but showing different detection profiles. The predicted unglycosylated form of CD44 proteins ranges from 39.5 to 81.5 kDa. In the MKN45 WT cells, CD44 proteins were detected in a higher interval of molecular weights (from 150 to 250 kDa), and CD44v9-containing isoforms were weakly detected in that range. For the MKN45 SC model, the global CD44 detection was concentrated to a sharper and single-band below 150 kDa, which coincides with the most intense molecular weight of CD44v9 detection. Regarding MKN45 MOCK, the profile of total CD44 resembles that of MKN45 WT, as expected, and CD44v9 is also weakly detected. Finally, the MKN45 ST6 shows a shift on molecular weight profile of total CD44, and the detection of an intense band of CD44v9, similar to the MKN45 SC model. Flow cytometry analysis was also performed for both surface and intracellular protein expression. The MKN45 SC has increased total surface CD44 and CD44v9 when compared to the parental cell line. In the MKN45 MOCK and ST6 models, only the CD44v9 surface increases in the ST6 cells. Furthermore, the surface expression differences observed were not seen in the intracellular fraction in the cell models. Given that the transcription of the CD44v9-containing isoforms is not altered, the differences in CD44v9 detection between the models may be explained by the differences in the protein glycoprofile.

### 3.3. Cancer-Related O-glycosylation Truncation Enhances CD44v9 Detection by a Monoclonal Antibody in Human Gastric Cancer Cells

**Figure 4:**
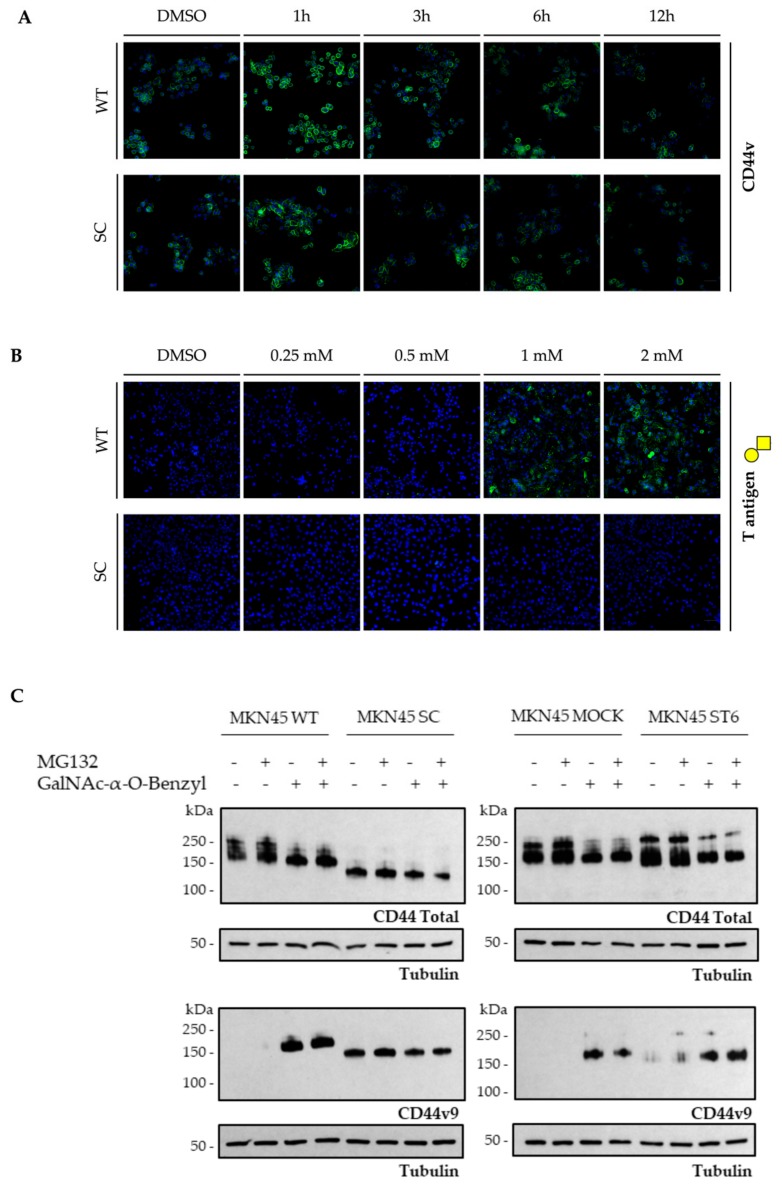
Absence of elongated *O*-glycan structures enhances CD44v9 detection. (**A**) Immunofluorescence analysis of CD44v upon gastric cancer cell line models treatment with the proteasome degradation inhibitor MG132 during different incubation times (1 h, 3 h, 6 h, and 12 h). The scale bar corresponds to 50 μm. (**B**) Immunofluorescence analysis of T antigen upon gastric cancer cell line models treatment with different concentrations of the *O*-glycosylation inhibitor GalNAc-α-*O*-benzyl. The scale bar corresponds to 50 μm. (**C**) Western blot analysis of CD44 total and CD44v9 in MKN45 glycoengineered cell line models, SC and ST6, and their respective control counterparts, WT and MOCK, in a context of proteasome degradation inhibition, *O*-glycosylation inhibition or both.

Glycosylation regulates many important cellular processes, affecting the structure of proteins and/or their interactions with other molecules [33]. Thus, the heavy glycosylation of CD44 could mask its antigens, rendering them inaccessible to antibody detection. Moreover, glycosylation may affect protein function, distribution, and even degradation [14,34]. Therefore, to investigate total CD44 and CD44v9 protein dynamics, we prevented protein degradation by inhibiting the proteasome with MG132 to evaluate CD44 variants’ presence in the cell lines. We also prevented *O*-glycosylation elongation using GalNAc-α-*O*-benzyl to assess the differential impact of truncated *O*-glycans in CD44 isoforms antibody detection. Since the *N*-glycosylation pathway is theoretically unchanged between the MKN45 cell line models, it will not affect the detection of CD44v9. We have confirmed this by digesting the cell models lysates with PNGase F (data not shown), and, as expected, there was a change in the molecular weight but not an enhanced detection of this isoform. A series of dilutions/incubation times were tested for MG132 and GalNAc-α-*O*-benzyl, and their efficiency evaluated by IF analysis of CD44v and T antigen expression, respectively (Figure 4A,B).

The concentration/incubation times were chosen according to the IF results, and protein extracts were used to perform a western blot analysis regarding the presence of total CD44 and CD44v9 (Figure 4C). All the MKN45 models showed total CD44 presence and a slightly increased signal upon treatment with the proteasome inhibitor when compared with the cells treated with DMSO only. Upon treatment with the *O*-glycosylation inhibitor, the total CD44 detection showed a sharper band around 150 kDa in all cell models except MKN45 SC, which unique band remained with the same molecular weight, below 150 kDa. When both inhibitors were combined, the two effects were seen: the decrease in molecular weight, except for the SC line, and a slight increase in protein detection.

After treatment with GalNAc-α-*O*-benzyl, the WT and MOCK cells showed an enhanced detection of a CD44v9 single band above 150 kDa. Hence, the pharmacological inhibition of *O*-glycan elongation on WT and MOCK cells mimicked the genetic models of *O*-glycosylation truncation on CD44v9 detection.

## 4. Discussion

CD44 is an important protein that has been implicated in tumor progression and resistance to therapy [10]. In fact, CD44 variants are overexpressed on malignant lesions and some of them have been regarded as promising diagnostic and prognostic biomarkers. CD44 variants interfere with specific cell transformation features, making them possible new targets for therapeutic approaches. In recent studies, bivatuzumab, a humanized mAb targeting CD44v6, underwent clinical evaluation in patients with head and neck or esophageal squamous cell carcinoma. However, its application was terminated early due to lethal toxic epidermal necrolysis, which halted further developments [35]. In fact, CD44 variants such as CD44v6 and CD44v8-10 are overexpressed but not exclusively expressed by tumor cells. Therefore, bivatuzumab is thought to also target the CD44v6 expressed on the skin epithelium [36]. More recently, RG7356 mAb, which targets the HA binding domain of CD44, was also used in a phase I clinical trial with patients with metastatic or locally advanced CD44-expressing solid malignancies. This mAb showed weak clinical activity, and the study was also terminated [37]. The targeting of total CD44 may interfere with T helper 1 cell antitumor function, which could weaken antitumor immune response [38].

As malignant transformation is associated with aberrant glycosylation, monoclonal antibodies directed to glycopeptide epitopes have been successfully developed [39,40] and have also been used in clinical trials [41,42]. In fact, cancer-specific glycosylation may add another layer of tumor specificity to proteins such as CD44. However, the CD44 glycosylation has been somewhat overlooked, as few studies have addressed this subject [8]. In particular, CD44v9 seems to have a major role in GC, and the combination with *O*-glycan truncation could present itself as a novel promising mechanism of malignancy. To dissect these mechanisms, we evaluated cancer-related CD44v9 expression in truncated *O*-glycan gastric cellular models. The global characterization of CD44 and CD44v9 was done in isogenic MKN45 GC cell line with two previously established glycoengineered MKN45 cell models. The SimpleCell and *ST6GalNAc-I* overexpression models are essential to study global phenotypes of cancer, including the glycosylation role in specific proteins, as they mimic glycoprofiles associated with aggressive phenotypes [14]. In the first, ZFNs were used to generate knockout cell lines for *COSMC*, the so-called SimpleCell (SC) model, in MKN45 and AGS cell lines [21]. This completely blocks *O*-glycan elongation, resulting in a homogeneous expression of Tn and STn exclusively. In the second approach, *ST6GalNAc-I* overexpressing plasmids were stably transfected in MKN45 GC cells [26]. In this way, the Tn glycan is preferentially used as a target by the ST6GalNAc-I transferase, favoring the formation of STn and competing but not disrupting the *O*-glycan elongation process (Figure 1A). The two models generate distinct Tn and STn expression, with ST6GalNAc-I overexpressing model showing a more heterogeneous STn expression profile as it still allows for a degree of *O*-glycan elongation. This agrees with mosaic pattern expression of the *ST6GalNAc-I* transgene often seen in overexpression models, likely caused by epigenetic modification of the transfected sequence [43]. This pattern of expression enabled the detection of differences in nondividing and dividing cells, which may show relevance in other work on cancer features.

*O*-glycan truncation does not significantly interfere with the global *CD44* expression profile, given by the comparison of MKN45 WT and MOCK with MKN45 SC and ST6 mRNA levels, respectively. However, when we look at the protein levels of CD44v9, there are clear differences among the models. These highlight the global challenges in the robust measurement of CD44v-abnormal patterns in terms of qualitative and/or quantitative expression. This evaluation has been done almost exclusively using mAbs directed against specific CD44 variant epitopes. Western blot analysis revealed distinct patterns of total CD44 and CD44v9 protein detection. It is known that glycans not only increase molecular weight but also affect protein stability and subcellular distribution [14,34], which can influence its function. We show that in a context of immature *O*-glycans, CD44v9 is enriched at the cell surface, which can be related to its oncogenic features [10]. Interestingly, intracellular staining revealed no differences, which disagrees with total protein extracts analyzed by western blot. This suggests that only CD44v9 with complete *O*-glycosylation is being differentially detected. To clarify, a western blot for intracellular and surface proteins fractions could be performed.

We present a model showing that complex and elongated *O*-glycosylation of CD44 hinders its western blot detection by CD44v9 antibodies, which could lead to an inaccurate readout from a variety of bioassays. Here, we demonstrate that the inhibition of *O*-glycosylation elongation from gastric cancer cell lines increases antibody-based CD44v9 detection. Immature glycans were shown to decrease protein stability and promote degradation [14]. To exclude differential degradation profiles in both scenarios of glycosylation, we combined an *O*-glycosylation inhibitor and MG132, preventing *O*-glycan elongation and protein degradation. CD44 has been shown to be increased when the proteasome is inhibited [44], which agrees with our results. This treatment does not promote CD44v9 detection whenever *O*-glycans are elongated. As demonstrated, CD44v9 detection by the RV3 mAb is only promoted when it is decorated with truncated *O*-glycans. The immunogens used to develop antibodies often do not consider the protein glycosylation, which can vary in the ultimate target of the antibody in the *in vivo* situation [45].

The RV3 CD44v9 mAb used in the present study has also been applied to evaluate the prognostic potential of CD44v9 in several cancer types. Kagami *et al.* showed that high expression of CD44v8-10, detected with the RV3 mAb in esophageal squamous cell carcinoma tissue samples, was a marker of poor prognosis [46]. In gastric cancer, Kodama *et al.* demonstrated that the expression of CD44v9, both at whole tumor and the invasive front, was associated with poor survival [47]. Although the immunohistochemical use of these mAbs may still be informative, our studies suggest that the huge structural variability of CD44 is conferred by not only its alternative splicing but also cell-specific glycosylation, which hampers immunodetection of some epitopes. Therefore, future analysis of RV3-based detection by immunohistochemistry should be done taking into account both elongated and immature *O*-glycan conditions. The possibility of lack of target protein recognition depending on glycosylation has recently been shown for other major therapeutic targets, such as PD-L1 expression-based stratification of patients. *N*-glycans were shown to inhibit PD-L1 antibody recognition, leading to a premature exclusion of patients for treatment [48].

Taking into consideration the record of clinical trials based on CD44 targeting, which revealed lack of efficiency in the clinical setting, it is needed to highlight that the glycoprofile of cancer-specific CD44v isoforms could be an answer for future studies and likely the success of such CD44-targeting mAbs. Our results demonstrate that the recognition of CD44v9 by mAbs is revealed to have major glycan and isoform dependencies.

## 5. Conclusions

Our results show the impact of truncated *O*-glycans in cancer-associated CD44v9 isoform variants. To our knowledge, this study shows for the first time that truncated *O*-glycans are involved in protein molecular weight and cellular distribution of the CD44v9 variant, and we demonstrated that immature *O*-glycan structures enhance CD44v9 protein antibody detection. This can have significant implications in clinical applications of this cancer-related isoform, as there is the possibility for misclassification of samples as false negatives.

The present work reinforces the utmost importance of taking into consideration glycan structures in cancer biomarker discovery and validation and that patient stratification for clinical trials should take advantage of individual protein glycosylation.
